# On the Treatment of Habitual Constipation

**Published:** 1884-01

**Authors:** Henry N. Josselyn

**Affiliations:** Late Interne of the New York Hospital; Omaha, Nebraska


### ARTICLE VII.

### ON THE TREATMENT OF HABITUAL CONSTIPATION BY OTHER THAN MEDICINAL MEASURES. By HENRY N. JOSSELYN, A.B., M.D., late Interne of the New York Hospital. Omaha, Nebraska.

I purpose, in the following pages, to do what I think I have not seen or heard done before. The fact that no previous attempts of the same sort have been made, is probably best explained by the very simplicity of the subject and the readiness with which the questions involved in it may be answered. My reason for attempting the task at this time, is the constant demand made upon physicians for a method of relieving habitual constipation without a resort to medicaments. What I shall say in this connection will be expressed in the simplest possible language, and with resort to as few technical terms as possible. This is done in the hope of making suggestions as to the course worth pursuing in explaining to the ignorant, matters with regard to which they are often in sore need of information.

It is both fortunate and unfortunate, that the chief attention of the medical mind has been given to the solution of the graver problems, which are suggested when life is threatened by disease. When these problems are pressed upon the attention it is with an importunity which is altogether terrible in its earnestness. If an aneurism is enlarging daily, there may be a fatal answer to the question: "What is to be done for its relief?" If a lung become tuberculous, it is of paramount importance to know what the future of such an accident shall be. But, for one person who has a pulmonary phthisis or a distending aneurism, there are a thousand who suffer from constipation. A disorder of this grade is popularly held in such light, that there are probably few students of medicine who would not feel justified in prescribing for its relief; while the druggist who could refuse to respond to the modest request made across his counter for a cathartic pill or other "opening medicine," would be generally set down as on the high road to a disastrous loss of patronage.

How to cure a common cold, how to relieve constipation, headache, and the ills that in popular estimation possess no greater gravity, are, it is true, questions daily answered and effectively answered by the practitioner; but the field none the less has been well contested by the charlatan, and by the man with a patent or a secret. It would be difficult to decide whose trophies are most numerous, but the chances are immensely in favor of the privateer. The "almanac," the brilliantly printed poster, the elaborately designed label, with all the other devices which wealth can command and ingenuity can execute, irresistibly affect the many. The windows and counters of the apothecary are more eloquent than the dissertations of the popular science publications. Indeed the most popular of all sciences in this day, seems to be associated with the art of delusion.

It might, perhaps, be regarded as an exaggeration, if it were said, that for the relief of what for want of a better term may be called chronic constipation, no single article of those recognized in the pharmocopœia, sold in the shops, or dispensed among the ignorant, suffices to secure the desired end. Nevertheless, this is a statement of exact truth. In the large majority of cases, it is by no means difficult to move the bowels. Yet it is precisely in the majority of all cases of chronic constipation that the common complaint is made: "I have tried everything and without success. My bowels can be evacuated once or more often by taking purgative medicine, but I am invariably left either in my former state, or even more constipated than before." The testimony on this point is uniformly alike. There are few physicians of practical experience who are not ready to admit that, while it is not generally a matter of difficulty to secure one or more alvine evacuations by the aid of medicaments, an attempt to break up the constipated habit is often a very serious matter, and even one sadly hindered at times by the very measures effectually employed to produce catharsis.

The larger number of the medicaments employed by the mouth for the purpose of emptying the bowels, operate either by stimulating the secretions of the intestinal glands, or of glands situated in physiological nearness to the latter ; or by a stimulation of the bowel action by either a direct effect upon its muscular envelope, or by an indirect effect through its nervous connections. It is idle to deny, that the system and the bowel, the nerve and the muscle, the liver and the gall-bladder, all tire after continual solicitation ; and, finally, be it ever so urgent, refuse to respond. It would be strange indeed if this were not the case. It would be a striking exception to a well recognized law, if this great complex excretory system did not, like all the other systems of the human body, cease after a time to utter its protest to the somewhat harsh and often brutal treatment to which it is subjected. The living muscle, after a time, will no longer contract when the electric current flashes through its fibres. The keenest appetite will pall after both satiety and surfeit. The jaded bowel no less than the jaded beast of burden, will in the end despise equally the lash and the spur.

It is quite unnecessary in this connection to enlarge upon the evils of habitual constipation as they are recognized in organs of the body other than the bowels themselves. As, in the state of fever, the entire economy suffers, so in its several systems, it may betray evidences of disturbance due to constipation, ranging from the slightest degree of perversion of function, to high grades of suffering. The brain, the sympathetic nervous ganglia, the blood-vascular system, the skin, the genito urinary tract, in brief, every region and every viscus may thus participate in the imperfect performance of the function of the great excretory conduit.

It is necessary, however, to freely admit, as all well-informed persons are willing to admit, that that condition which might be called in one person habitual constipation, may in another be consistent with perfect health. Every physician has met persons, of whose excellent physical condition not the slightest doubt could be entertained, in whom each ingestion of food by the stomach was followed by an alvine evacuation. Every physician of experience has also known individuals presenting all the appearances of sound health, whose rectum was habitually emptied but once every three or four days, and even but once in the week. This wide range of performance of function with strangely separated limits on either hand, is peculiarly true of the human animal. We can explain to a degree both its conditions and its possibilities, when we reflect upon the difference between the Esquimaux living in a temperature from twenty to seventy degrees below zero, his stomach stuffed with blubber and his skin protected by thick, furry garments, and the naked Fellah of Egypt, subsisting without difficulty on a little rice and a handful of dates. A decision as to the question whether, within these limits, the function of the bowel is or is not properly performed, will depend of course upon the existence of subjective or objective symptoms which indicate disturbance of the general economy.

The causes of the form of habitual constipation, to which attention is here particularly directed, are both numerous and varied. The causes fully recognized in the text-books treating of the subject, need not, of course, be here fully described. The derangement of nerve, of muscle, of secreting epithelial pouches, the direction of the bodily energy in excess to other organs (hyperidrosis, enuresis, etc.). the lack of intestinal juices, the absence of peristalsis, the effect of ingesta, including those articles having a toxic or narcotic effect—no need, indeed, to enumerate all these. For the present purpose it will be more pertinent to inquire what conditions lie behind all these others; what causes the failure of the secretion, the tiring of the nerve, the weakness of the muscle. Is there a condition, or are there conditions, lying at the base of this complexus of symptoms, by whose recognition we may reach remedial measures capable of better effect than scammony, mercury, castor oil, or the lately suggested substances whose very names would have made our fore-fathers in medicine stare with unfeigned astonishment?

Such a condition does exist. We all in some degree recognize it; though, like everything else that lies within the range of a common experience, it is apt to be ignored. Few swimmers speculate on the specific gravity of their bodies and of their limbs. Of all who look upon a tree, but few will detect the exact outline of its leaves. And so the conditions imposed upon humanity by its modern civilization, especially upon the men and women congregated in cities and towns, furnish the sole explanation that is needed for our present purpose. The Romans who formed the legions that Cæsar led into Gaul; the rank and file of the regiments that take the field to-day; the American Indian on the plains; the native African sunning himself lazily on the banks of the Congo River—these have no troubles of the kind recognized every day in the hard-worked seamstress, the over-taxed man of business and the fashionable woman of the period.

It would be impossible, in these limits, to fully discuss the several elements which together constitute the main source of this disorder prolific of so many others. It will suffice here to briefly touch upon a few of the most important among them.

First, both in importance and frequency of effect, may be named, the failure of sufficient bodily exercise.

It may well awaken surprise to hear it soberly stated that Americans, who are so often accused by their English brethren for the restlessness of their activities, actually suffer from a lack of physical exercise. It is nevertheless to a great extent true. The day-laborers and hand-workers of this continent are not very largely Americans by birth. The men born on this soil who live in its towns and cities, are, during the day, chiefly occupied at the counter, at the desk, in the ware-rooms, or "on 'Change." Exercise of a certain sort they get, wearing equally upon mind and body, but never of the generous quality which is his who ploughs, who digs, or who lays brick. The constipated man, to whom we refer, drives to and from his place of business in his own equipage, or bestows himself within the offensive compass of a city railway car, where persons of both sexes are huddled together morning and evening in disgraceful and even dangerous proximity. During the day he sits at his books or stands at a desk, his muscles dwindling, his blood propelled through his languid veins more feebly with every year. He would look upon a walk of ten miles as a punishment rather than as one of the readiest sources of the delight and pleasure of healthy living.

When we turn to his wife, his daughters, and his sisters, the picture in all ranks of society is a wretched one from the physical point of view. Their representatives in the lower classes drag out joyless days in the boarding house, from which they issue only to visit the shops and the apartments of their friends, or to seek the unreal and unhealthy attractions of the theatre. If they are occupied with the cares of a domestic establishment, or, to use their own language, the burdens of "house-keeping," they have neither time nor ambition for much beyond. Aside from the inevitable excursion to the shop, the market, or the house of a neighbor, they neither take nor crave any exercise in the open air which can be deemed sufficient for the purposes of health. Many unmarried women, employed as seamstresses, clerks, factory girls, shop women, etc., exercise, without doubt, to the point of weariness within the four walls where they are engaged in labor, but without that measure of healthful activity of the body and limbs which they really need. In the upper ranks of society, the days are spent in an almost pitiful idleness. The occupations of the drawing-room or "parlor," of the piano, of the social call, of the drive in the carriage when the weather is fair—these are well nigh the sole employments of the body which they at any time either undertake or even dream of undertaking. They would, for the most part, regard him as guilty of either eccentricity or folly, who would intelligently direct them to take a sufficient amount of exercise by pedestrianism or by exercising in any other suitable way so as to arouse their viscera from an habitual torpor.

Even their babies are wheeled indolently about the streets in delicately upholstered "baby carriages" on wheels, instead of being carried about in the arms, an exercise which not only employs the muscles of the mother, but which makes no little demand upon the muscular vigor of the child. Many of the infants in our large cities would be far better in health if their carriages were destroyed and they themselves were left where nature first put them, in the arms of their mothers and nurses.

Are these women they who suffer from habitual constipation? The gynecologists, consulted about so many ills of the uterus and other pelvic viscera, can give a convincing answer in the affirmative. Constipation is, without question, the rule and not the exception in this class of patients. Its long and dreary history, including the occurrence of "blind" and bleeding piles, prolapse of womb or bowel, leucorrhœa and other pathological vaginal discharges, pain, aching, an indescribable sense of languor and weariness, often complicated by pregnancies when the physical state is utterly unfit for child-bearing and the mental condition equally unfortunate for the obligations of the parent,—all this is abundantly well attested in the pallid, sallow-skin, flattened bosom, flabby figure, and general appearance of the worn woman, the burden of whose complaint occupies so large a space in the records of medicine. I have dwelt somewhat more fully upon the afflictions of women, simply because the picture presented by them is rather more vivid and instructive than in the opposite sex. The indolence, voluntary or enforced, of so many women; the tender provision for their wants, usually accorded with generous fulness by their natural protectors; and the fatal ease with which their lower pelvic basin tolerates, without the production of distress, a distended rectum, these are sufficient reasons why they should suffer above others.

The second of the great causes of constipation to which I desire to call attention, lies in the numerous errors committed with respect to ingesta. I shall briefly speak of these as they naturally arrange themselves into errors respecting food, drink, and medicaments.

It would not be difficult to classify the common methods of food preparation in this country, by separating them into four distinct categories. In the order of their demerit they may be illustrated by the following types:

The lowest and most abominable can be best studied in the poorest class of western hotels and in the cheaper lodging houses both of the city and country. It would be difficult to say whether ignorance, greed, moral depravity, or an inherited failure to beget repugnance to filth, lies at the root of its enormity. The food here is always placed before the eater without apology for an attempt to make it seem attractive to the eye. Of its effect upon the palate, perhaps, the less said the better. It is cooked in the crudest of methods by ill-paid and ignorant persons of either sex. With respect to its quality, before it has been subjected to any culinary process, the philosopher would look upon it as one of the amazing and incongruous puzzles presented to his observation in this land of fertile fields, grazing herds, abundant markets, and cheap railway transportation. Disjected fragments of ill-favored meat, fried and swimming in an indigestible semi-liquid grease; solid wedges of sour corn meal; livid disks, compounded of buckwheat flour and water; articles such as these are daily set on tables before commercial travelers and day laborers in many parts of this country.* It is after his stomach is filled with such food as this that the victim turns with relish and an instinctive need for the promotion of digestion, to a glass of spirits or to the cheapest grades of tobacco for use in either his pipe or his mouth. The bar-room

---

*I cannot resist the temptation to compare the condition of the food placed before the middle class of our people in many parts of this country, in this, the evening of the Nineteenth Century, with the condition of our English ancestors over five hundred years ago. In the year 1336 the Parliament of Edward III., in consequence of the excesses and expenses of the tables of not only the great but also of the "lesser people" in the realm, enacted a law that "no man, of what estate or condition soever he be, shall cause himself to be served, in his own house or elsewhere, at dinner, meal, or supper, or at any time, with more than two courses, and each mess of two sorts of victuals at the utmost, be it of flesh or fish, with the common sorts of pottage, without sauce or any other sorts of victuals. And, if any man choose to have sauce for his mess he may, provided it be not made at great cost; and if fish or flesh to be mixed therein, it shall be of two sorts only at the utmost, either fish or flesh, and shall stand instead of a mess," etc. 10 Ed. III., Cap. 3. Harrison, writing of the English people but a few years later in the same century, describes the "great shins of beef" eaten by the "comyn people" with "mutton veal, lamb, pork, whereof one findeth great store in the markets adjoining; besides souse, brawn, bacon, fruit, pies of fruit, fowls of sundry sorts," etc., in consequence of which they did "fare commonly so well as the king."

If any reader of these lines concludes that I am laying too much stress on this point, I pray him to read this description and then to put up at the first country hotel he finds one hundred miles beyond any town having fifty thousand inhabitants, in one of the Northwestern States.

and the shop of the tobocconist are far wiser than to display their wares in the unattractive and even disgusting setting of the cheap lodging-houses. The inmates of the latter will often admit that they seat themselves at the dining table chiefly in order to secure a distention of the stomach sufficient to make acceptable the stimulants that are to follow.

The second of the methods referred to above, is exemplified in the better class of boarding houses, and the second-rate city and country hotels. In these the food is usually prepared by female servants, often ignorant themselves but acting under the instructions of a mistress or head cook. The meals are served with a decent regard for cleanliness; fried abominations are seldom seen; there is a fair variety of roast meats; fish is cooked after a fashion, and some delicacies appear upon the table. But there are grave errors at every meal. The table companions are seated closely together; their time is limited; conversation is subordinated to the gratification of appetite; the meal is hurried; and the great American pie, manufactured by wholesale, is always patronized. Iced-water is drunk in large draughts by the eager and hurried feeders. The bread is invariably poor. A dish of properly cooked vegetables is among the greatest of rarities; and the potato, which judiciously treated in its jacket is one of the precious luxuries among the poorest of trans-Atlantic nations, is transformed into a water-logged and altogether wretched travesty of its better self.*

---

* "If there is any truth in the proverb, 'Tell me what you eat, and I will tell you what yon are,' it is amply illustrated in the new world. The American does not eat, he simply fills himself; his sole desire is to satisfy hunger, and, if he can secure quantity, quality is of little importance; hence one frequently sees him ingest a lot of food standing up, or scarcely seated, at a counter. There are fixed hours between which meals may be had, and food must be taken at those times, whether one may be hungry or not. A despotic, all-powerful public opinion, which no one dares defy, decrees the hours for eating. The meals aré always begun with a square block of rancid butter, having au odor of margarine. A pause of fifteen to twenty minutes gives time to attempt the digestion of this compound, and then you find yourself surrounded by a variety of little dishes which you expressed an intention to eat. It matters not that they become cold, for do you not see around you 'des misses, des ladies, des gentlemen's,' eating watermelon with chicken, and currant jelly with cutlets and salad! Behind your chair is one negro who fans you, and another who puts ice in your glass. Americans have a number of religions, and but one sauce; that is like glue, by which otherwise good meats are ruined. Their eggs have a queer, stale animal flavor, which is supposed to result from the hens being fed upon meat that they may lay more. Corn is a favorite article of food, and, though eaten in various ways, is most often boiled on the cob. Three or four of these cylinders are not too much for a lover of this cereal, who will gnaw off the grains with his teeth, and presently leave nothing but 'la carcasse.'"—(M. Poncet), *The Medical Record* Dec. 1, 1883.

Of the third type little need be said. It is well shown and quite too well paid for at such places as Delmonico's in the city of New York. The high prices which it commands, may be counted in some sense as a measure of the greatness of the demand for, and the paucity of the supply of such elegance. Beyond the reach of all save a wealthy few, the expensive and attractive adaptation of the skill of the French cuisine to American needs, may be made to contain the elements of relief for certain forms of dyspepsia and constipation.

But, lastly, the highest type of excellence for the table of the native American should be found in his own home. Here he has the opportunity of so directing his household that the health of all its members may be subserved by the ingestion of properly selected and suitably cooked articles of food. Of the grievous errors here committed, in ignorance, in recklessness, or in haste, space would fail to tell. If here are the triumphs, here are also the failures, which the wise physician has occasion to regret. At its worst, however, there is this in its favor,—the table is set in the midst of a home; the mistress herself purchases at the market rates and does not pay another to do it for her; she can, in an emergency, correct the blunders of a worthless cook, and even avert the fate of an omelet doomed to destruction in less skillful hands. The wholesome habit of preparing food for one's self is much better understood abroad than in this country. Every undergraduate of an English University can brew a cup of tea for himself in his own apartment, and, with the same limited accommodations, extemporize for his friends a modest but appetizing repast. Every Frenchman understands the mystery of the *pot-au-feu*, and can infuse coffee in a way that will bring the water to the mouth of him whose nostrils are reached by its aroma. Has any reader of these lines by this time concluded that I have drifted far from the subject to which it was promised that these pages should be devoted? If there be such, let me assure him that I am right in the heart of my theme. A common experience may be cited as the best proof:

Take, for example, the man who complains of being "bilious." The symptoms which, if he be intelligent, he will describe in detail, such as vague pains, anorexia, and even distaste for food, mental hebetude, constipation,—these we may be sure are all real, and not the offspring of any hypochondriasis. The usual remedy for such a condition is a blue pill or some other dose of cathartic medicine. This will usually ensure relief within forty-eight hours, an interval which in many cases, however, will be passed with a very considerable degree of discomfort.

But the curious alchemy of nature laughs at the clumsy methods of art! This man, with a stomach distended with gas and an unladen colon, is unexpectedly invited to dine with some friends. He concludes to defer his pill and to accept his invitation. Once at table, his several senses are gratefully stimulated by enticing odors that exhale from appetizing dishes spread upon a luxurious board. He is too wise to abuse his privilege as a guest. He eats, he drinks, he smiles, he converses, and at last he laughs! His stomach, that aforetime refused its gracious offices, now gladly consents to do its part. Word is speedily sent along the line that work is to be resumed, and all the agents of digestion bestir themselves. Pent up bile seeks the duodenum; the pancreas participates in the general activity; the solitary and agminated glands below answer to the call from above. On the next morning, the guest, to his astonishment, enjoys an ample movement of the bowels; escapes from the bondage of yesterday's distress, and even acknowledges a keen desire for another meal. Here is a charm, whose magic no pill, no draught, no dose of salts can rival. We all know how this is, but we do not all learn and remember its lesson. To secure the highest digestive effects, the food taken into the stomach must be of wholesome quality, acceptably cooked, and tastefully served. There is a moral side, even, to a constipated bowel.

The vexed and mixed question of the influence of fluid ingesta upon the health has been almost hopelessly confused in this country by well meaning but often misguided people in a controversy spreading over questions of morals, religion, temperance, prohibition, total abstinence, and who can say what beside! *

* "I am against dealing with a question of this nature (the liquor question), affecting the interests of so many people, by what you may call a hurricane. That is fit only for times of revolution. I should like to deal with it in a more just and what I call more statesmanlike manner, according to the legislation that becomes an intelligent people in a tranquil time."—*Hon. John Bright.*

"It would be impossible for any set of men to manifest greater bigotry and intolerance

It was, I believe, Lord Macaulay who first declared that the soldiers of an army acquiring by conquest a wine-producing country, always began with the grossest intemperance, and always concluded by the most moderate indulgence in the use of wine, and that drunkenness was least common among the inhabitants of grape-producing districts. However disputed his position, it is certain that education has much to do with sentiment on the subject of the use of wine, spirits, and beer. There are very few clergymen of any church in Europe, who would like to be seen drinking spirits, but there are far fewer who do not regularly and habitually make use of wine or beer. At a *fête champêtre* given the members of the National Evangelical Alliance at Basle, in the year 1879, it was reported in the public press that "beer and wine flowed like water"; yet, fully seventy-five per cent. of those who were present were clerical delegates and their wives.

The wise physician will always be a citizen of the world and refuse to share the prejudices of place or season. No one better than he can tell the tale of wretchedness, poverty, disease, and shame, that may be charged to the account of intemperance in drink. It is he who visits those moral and physical wrecks, which in every community serve as dismal warnings of the stress of temptation, and of the shoals where so many victims are stranded. He has watched, too, long before the miserable end, the repeated failures to escape once more upon a smoother sea. If he has not dropped a plummet into those depths wherein have fallen husband, wife, and children, he has had but an imperfect experience of the community in which he essays the practice of his profession.

But he is also well aware of the fact that honest lives, healthful lives, and sweet lives, are consistent with the habitual use of wine and beer. He will counsel no man to make use of these in the absence of sufficient reason; but he will frown on no man

---

toward all who have seen fit to differ from them on moral and religious measures, than have characterized those zealous and thoroughly well meaning reformers who, through various organizations, have assumed the custody of this (temperance) question. Editors who have undertaken to discuss the question independently—as they are in the habit of discussing all public questions—have been snubbed and maligned until they have dropped it in disgust, and turned the whole matter over to those who have doubted or denounced them."—*Dr. Holland.*

who, being in the possession of good health, may choose to use them in prudent moderation.

What good the temperance reformers have wrought in this country, has been unmistakably mixed with one broad evil. They have made the temperate use of wine and the lighter malt liquors so disreputable in the eyes of the many, that the practice has been driven from more respectable surroundings to the bar-room, the saloon, or the dining room closet. In what American boarding-house can a man or a woman regularly open with each dinner a bottle of Bordeaux or Burgundy, without at once becoming the subject of a merciless social ostracism? How many gentlemen can indulge in a glass of champagne in the company of ladies in any public resort, without awaking the suspicion of all beholders with respect to the character of each member of the entire party? This is more than intolerable. There is a flavor about it of actual indecency. It is a part of that ostracism which has driven every man who is in need of even a glass of sherry to the malodorous precincts of an American bar. With reference to the whole matter, it may be truthfully said that, all practices connected with eating and drinking that cannot be generally approved and adopted in the company of gentlewomen and well bred men, are social evils that will in the end become social vices. When there shall be in this country a healthy sentiment that approves and encourages the temperate

* "Wine-drinking populations have keener, livelier wits than those who use other beverages. It is proved by long experience that the pure juice of the grape sustains the force of activity of the brain. The poets who, from age to age, have sung the praise of wine, were not wholly either deceivers or deceived. In the lands of the vine, where the plant is looked upon as a nursing-mother, men do not injure their health by drinking, but in the colder North where the grape can never ripen, the deaths from intemperance are frequent Bread and wine are almost pure gift of Nature, though both are prepared by man after the old traditional ways. These are not poisons, but gin and absinthe are poisons, madness poured out from a bottle! Kant and Goethe loved the pure Rhine wine, and their brains were clear and vigorous to the utmost span of life. It was not wine that ruined Burns and Byron, or Baudelaire, or Alfred de Musset.

That honest Northern drink — beer, deserves our friendly recognition. It has quite a peculiar effect upon the nervous system, giving a rest and calm which no other drink can procure for it so safely. It is said that beer drinkers are slow, and a little heavy; that they have an ox-like placidity not quite favorable to any brilliant intellectual display. But there are times when this placidity is what the laboring brain most needs. After the agitations of too active thinking there is safety in a tankard of ale. Wine-drinkers are agile, but they are excitable; beer-drinkers are heavy, but in their heaviness there is peace. In that clear, golden drink which England has brewed for more than a thousand Octobers, and will brew for a thousand more, we may find, perhaps, some explanation of that absence of irritibility which is the safe-guard of the national character, which makes it faithful in its affections, easy to govern, not easy to excite to violence."—(*Philip Gilbert Hamerton*)

use of wines and malt liquors in public, in the presence of women and children, and in the view of all the world, there will be more genuine courtesy in our manners and much less hypocrisy in our characters.

The important bearing of this question upon the digestive tract can scarcely be disputed. Every Frenchman looks with some justification upon his *vin de pays* as the great aid and promoter of his digestion. When in bed, prostrated by an illness producing inability to assimilate food, the first article he craves is his *eau trempée*, toast moistened in a weak mixture of wine and water. It is a marvelous thing to watch him when he is in his usual health and at dinner. Slowly, very slowly, he sips his glass of Bordeaux, mixing it well with the food, which he takes in small morsels, into his mouth, all well and thoroughly masticated before entering the œsophagus. No iced water for him, if you please! He would not chill his stomach with such an Arctic libation for all the treasures of the Louvre. He who has not seen and noted the leisurely performance of a French gentleman at his dining table, has never mastered the rudiments of gastronomy, and has yet to learn the alphabet of assimilation.

Perhaps the reverse of the picture may be well studied for a moment. Our American understands perfectly the method by which he proposes to satisfy his physical cravings. He desires a given effect, and that with as little trouble and delay as possible. Wine and malt liquors are for him quite too slow in their operation; and, besides, 'let them say what they will, a native Kentucky whiskey is more suited to his taste than any wine made.' When he wishes it at all, he wishes it served promptly, for example, in that brief respite when the Stock Board has ceased to resound with its scarcely less stimulating yells. He wants it strong; he wants it speedy; he wants it "straight." Even the spirit-drinking Englishman does better than this, for he satisfies himself by pouring a small quantity of brandy into a large quantity of water, which he drinks only in this state of large dilution. The American cannot afford to outrage public opinion since he may be a candidate for Congress in a few months. Stranger things have happened! Accordingly he slily slips around a corner or into an alley, where he calls for his glass. Once in his hand he literally pours the fiery stuff down his throat. If there happens to be no special haste, he repeats the dose in the company of his friends. Small wonder that this man's digestive tract is soon in that condition when all the pepsine of all the swine in the corn fields of Kansas cannot work the miracle of its cure. And yet this poor, ignorant fool, the woes of whose digestive canal we have all studied, from his shrunken liver to his bloated mesentery, is held up to the world as a fair sample of that gracious company which includes the wisest, bravest and kingliest of mankind in all ages since the first cultivation of the vine! It includes such men as Horace, Constantine, Virgil and Cicero, among the ancient Romans; such men as Coleridge, Wellington, Milton, and the lion-hearted Richard, of England; the illustrious line of the philosophers and savants of France from the day of Charlemagne to that of the great Napoleon; the splendid names which the German empire has lately given to the world, whose superb acme is Prince Bismark himself; and even the reformer Martin Luther, whose four hundredth birthday was celebrated but a few weeks ago. I dismiss this part of my subject with the apophthegm, that while the weaker alcoholic beverages can undoubtedly be so used as in the highest degree to promote assimilation and digestion, not one American in a hundred has the slightest conception of how this is to be done.

With regard to the consumption of drugs by those who seek to be in this way relieved of constipation and its attendant ills, it is to be remarked that they who adopt such a practice usually suffer before the end is attained. The consumption of rhubarb is possibly less common now than in the days of our grandfathers, when the chewing of the root, both in public and in private, was the solace of many unoccupied moments. The unfortunate tendency of the people of our country to believe in the assurances of the vendor or proprietor of patent medicines, has deluged the country with every form and variety of cathartic pills and purgative medicine. As to the constituents of their nauseous compounds, it may be well said in the language of one of the oldest as well as one of the wisest pharmacists in the United States: "There is money in them all and nothing else." These are properly considered among the causes of constipation. The dele·erious influence they exert on the alimentary canal by their drastic, stimulating, or cathartic effects, is due, not merely to the unfortunate ease with which the dose may be procured of any druggist and swallowed as often as the slightest whim or uneasy sensation may dictate. It is based also on the fact that since there is no limit to what the manufacturer may choose to put into his compounds save that set up by his own conscience, there is a constant and almost irresistible temptation for him to substitute coarser or more drastic drugs, such as caballine aloes, Glauber's salts, croton oil, etc., for the better articles which at the first aided in gaining a reputation for his nostrum. "Aided," I say, for there can be no question but that a sum of money judiciously expended in widely advertising a solution of brown sugar in water, for any purpose whatever under the sun, will pay handsomely for the investment just so long as there are more fools than wise to read and believe in advertisements.

If this can be said of cathartic remedies that are advertised to relieve constipation, what words can do justice to the disastrous habits into which have fallen so many otherwise sensible persons of taking drugs for the purpose of gaining other effects, but who in the end accomplish this unexpected and unwelcome result. Such are they who have accustomed themselves to taking a few grains of chloral or the bromide of potassium on retiring to bed "for the sake of securing a night's rest." Others have been swallowing all sorts of compounds for the purpose of "cleansing the blood." Here, for example, is an extract of "clover blossoms." What an innocent name! Who could refuse to taste of that from which the honey-bee has gathered its luscious store! Yet, curiously enough, it is they who have been making use of the so-called "vegetable purifiers of the blood," who display eruptions upon the skin not to be distinguished from those produced by the iodide of potassium. Some of the "sarsaparilla" and other proprietary articles are known to contain still more harmful ingredients. It is one of the inscrutable paradoxes to be recognized in human nature, that some of the greatest cruelties it has ever practised have been committed in the name of religion; and that a man will actually pay a physician for telling him how to be rid of an ailment which he is at the same time daily engendering or aggravating by an act of his own folly and at an expense to his pocket.

Lastly, the immoderate use of tobacco, though not properly included in the list of ingesta, may be named among the common causes of the ailment under consideration. The subject is one that will, probably for a long time to come, be the occasion of a controversy in which the belligerents on either side display as much zeal untempered with discretion as in the warfare upon alcohol. But here also the wise physician will attempt to take a sound position, without exposing himself to the serious charge of prejudice. The consumption of tobacco has steadily and regularly increased in all countries since the date of the discovery of the uses to which it may be put; and it is safe to predict that it will always be prized by the human family. Revolutions, it is said, never go backward. The "divine weed," as Spenser first named it, a solace for the dispirited and a support for the weary the world over, first grasped in the needy hands of the contemporaries of Sir Walter Raleigh, will never be relinquished by their descendents while toilers can rest and the care-worn gather comfort for the morrow from its grateful incense. To-day, no less than when the words were first set down by Kingsley, is it recognized as: "A lone man's companion, a bachelor's friend, a hungry man's food, a sad man's cordial, a wakeful man's sleep, and a chilly man's fire; there is no herb like it under the under the canopy of heaven."

But Americans are nothing if not extremists, and, therefore, he who refuses to raise his voice against the damage inflicted by their excessive and extravagant use of tobacco, is not discharging his full duty as a physician. Of its injurious effects upon the young we need not here speak at length, since these do not form the class which chiefly suffers from constipation and its attendant ills. Gorgas, in his late report* upon an experiment made on a sufficiently large scale in depriving of tobacco the cadets of the

* Report of the Secretary of the Navy, being part of the Message and Documents communicated to the two Houses of Congress at the beginning of the Second Session, 47th Congress, Washington, 1882. Report of the Surgeon-General of the Navy, page 612. Report of Medical Inspector, A. C. Gorgas.

United States Naval Academy, well demonstrates the physical improvement which such abstinence can work in the case of maturing youths. Drawings made by the same person when completely free from the influence of tobacco, and also after excessive smoking, showed by comparison differences which even the least expert could recognize at at glance. It is somewhat remarkable that aside from the tremor and muscular weakness betrayed in the drawing made by the smoker, one could also recognize a subtler effect produced upon the brain and the eye of the artist as a result of which he either did not understand or failed to perceive the direction of certain lines and the form of certain objects.*

No experienced practitioner needs to be told again of the failure of co-ordination of muscular movements resulting from the injudicious nse of tobacco; or of the "tobacco-heart," first well described by Mitchell, and since recognized in enormous proportion among soldiers, sailors, car-drivers, prison criminals and others of like class, who throughout the entire day can gratify the continual temptation to keep the mouth filled with chewing tobacco of the cheapest grades.

But, to refer more explicitly to the subject in hand, what shall we do with a fact first announced by a no less keen observer than Trousseau, that an after-breakfast cigar will often ensure for the smoker, and that quite promptly, a satisfactory stool? This is a perfectly true statement, and there are thousands who have verified its accuracy. It is also true, in the case of many persons who suffer from torpor of the digestive tract, that the habit of smoking a post-prandial cigar serves to promote peristalsis, to stimulate secretion, and thus to improve the performance of many functions essential to the best nutrition of the body. These admitted facts, however, do not stand in contradiction with the general laws whose violation by intemperance results in harm. For one who is sufficiently interested to investigate this matter,

* It is scarcely necessary for a man of ordinary observation to be told that the practice of smoking tobacco is consistent with the highest intellectual attainments. For example, a late sketch of the famous poet-laureate of England, Alfred Tennyson, from the pen of Mrs. Ritchie, gives us a picture of the greatest author of English verse, "smoking in a top room in Eaton Square some Durham Tobacco which Lowell had given him."

The regular breakfast, in all seasons of the year, of the profound thinker, Kant, was a cup of tea and a pipe of tobacco.

it is astounding to count up the number of men who smoke six, twelve, and even more cigars, or a package or two of cigarettes daily ; men who, when not engaged in eating and drinking, are teased by nerves that quiver for their accustomed narcotico-stimulant five minutes after its withdrawal ; men who smoke in the morning before breakfast and at night before getting into bed, it matters not whether the stomach be full or empty ; men who practically never escape from an atmosphere charged with the products of tobacco-combustion ; whose food and drink cannot but be more or less contaminated from the same source. For these, and for those, too, whose hygienic errors are far fewer than theirs, an habitual torpor of the liver and of the secreting glands of the intestine unquestionably results, accompanied or not by the cardiac and cerebro-spinal symptoms to which I have referred.

A simple rule can be applied to tobacco-usage, whose scope will properly include a long list of luxuries. The man of mature years who uses tobacco in moderation as a luxury only, or as an occasional means of promoting digestion, or as an often effective measure for preventing the accumulation of superfluous flesh, or for other purposes unconnected with the continual yielding to the solicitation of his nerves, should generally be indulged in the privilege. But he who has become the slave of an uncontrollable appetite for tobacco, must be encouraged, scolded, commanded, and aided, in the effort to escape from his bonds. There is peril to his health in every week of delay, and the great army of tobacco users for the best part of a century will confirm the truth of the warning.

If one will comprehensively and without any prejudice, review for himself the entire field which in these pages has been briefly visited at a few points, he can thus form some idea of the picture presented by the people of this country to foreigners, who look upon us with critical intelligence and a not unfriendly eye. To them, we for a brief moment or two, expose those features which we look upon in each other for a lifetime. Our vision, dulled by uniformity of impression and absence of just comparison, accepts with incredulity the testimony of other eyes. Before the world, we parade our juiceless men ; our slender and too graceful women ; our cheeks, equally lank whether they compress a quid or make a vacuum for the draft of a cigar ; our sallow skins, sympathizing with an inactive liver and an undistended spleen ; our nervous systems, equally ready for original invention and for the development of new types of nervous diseases ; and we wonder that such visitors to us as Mr. Herbert Spencer and some other of his distinguished countrymen, can speak of us as they do.

The whole subject is well illustrated by an incident which occurred last summer. An excellent representative of native born Americans, the direct descendant of a line of men who for two hundred years have digested the food grown upon this soil, a fair type of our race in his social position, wealth, intellectual acquirements, and . . . . . . poor physique, being in London, consulted an eminent British practitioner about his health. "Doctor," said he, "I believe that I am bilious, and need some medicine to get rid of my bile." "Why, bless your soul," cried the burly representative of our profession, as he gazed at the sallow features and thin cheeks of our eminent American, "the very thing you need is a larger quantity of really good bile ! "

My subject has already enticed me into a far wider digression than I anticipated at the outset. If I have, in the preceding pages, correctly set forth the truth, there should be but little left to be said respecting the therapy of the disorder under discussion. He who has recognized the cause, is in the best position to relieve the condition. It will be proper, however, to conclude with a hasty review of the measures which may be employed for the relief of habitual constipation and its results, to the exclusion of medicaments.

First, then, sufferers of this class need more systematic bodily exercise. Too many persons of both sexes, living in our large cities, make use of the street railway cars. Many a merchant, and many of his subordinates too, seated or engaged throughout the day in sedentary employment, would be far better both in health and in pocket for the walk, morning and night, over the few miles which separate their lodging-houses from their places of business. Women, more particularly, should make a practice of walking a given number of miles every day, attempting at first short distances only, and gradually pushing their excursions out toward the suburban towns and the open country. For those unable to spend the time demanded by these excursions, the gymnasia are open to both sexes; as also are the swimming baths and the riding schools. Many young women have been safely piloted by our wise gynæcologists through a perilous puberty to the richness and fullness of healthy womanhood, after a resort to equestrianism. To be able to ride well, is one of the most graceful, invigorating, and delightful of accomplishments for woman; and scarcely less can be said of a long list of outdoor recreations, including swimming, skating, lawn tennis, croquet, etc.

A large number of men, too, who are daily trundled about in their phaetons and coupés, would be much better if they were made to mount one of their own horses. Fortunately, horsemanship in this country is every year gaining in popularity with both sexes, since its possibilities have been both created and enlarged by the construction of public parks set aside for recreation, within or near many of our large towns.

Unfortunately, equestrianism, both in the riding schools and with the resources of a private stable, is a more or less expensive luxury quite beyond the reach of many. The same may be said, though to a less degree, of the invaluable aid rendered by the gymnasia, which also are often inaccessible by reason of distance, or are so crowded at certain hours of preference, as to be undesirable for the purpose in view.

The domestic apparatus, however, adapted for use in one's home, is within reach of many, and can be made to accomplish a great good. Still, the Indian clubs and dumb-bells are simpler and less expensive. *With a single dumb-bell*, that may be purchased in any city at an outlay of but a few shillings, *every muscle in the body may be exercised*, *strengthened*, and *actually made to increase in size.* As the proper method of employing this useful instrument is not very generally understood, I propose to give specific directions as to the manner of using it so as to secure the best results.

One, or a pair, of dumb-bells may be used, the weight of which may be determined by the number to be employed in exercise, and the strength and years of the individual for whom they are intended. It is better to begin with a small weight and a single dumb-bell, till the muscles of the body are somewhat habituated to its management. An adult male can advance to the regular use of a dumb-bell weighing thirty pounds; while women generally should not begin with one of more than ten pounds weight. One should exercise only on an empty stomach, in a well-aired and cool apartment, with a single loose covering for the upper part of the body, such as a woolen under-garment. The iron handle of the dumb-bell should be covered with soft leather in order to protect the palms.

Standing at ease in an erect posture, with the feet slightly separated and the dumb-bell lying between them, the body is strongly flexed by the sheer force of the muscles of the trunk and the lower limbs, while the arms depend loosely at the side. When one hand is within reach of the dumb-bell, the latter is grasped and merely held firmly while, by the aid of the muscles of the trunk and lower limbs, the body is brought to the erect position again, the hand which holds the dumb-bell being thus carried to the side, its arm in full extension. Care should be had not to assist this movement by resting the free hand upon any part of the person, such as the knee of its side. Then slowly, and without taking advantage of the momentum which might be acquired by a jerk, the arm supporting the dumb-bell is brought into a state of extreme flexion, till the forearm is nearly parallel with the arm, and the hand which grasps the dumb-bell is turned upward. By the same slow process, and by the assistance of the deltoid and scapular muscles, the dumb-bell is elevated above the head to the extent of the full reach of the arm. It is lowered to the ground as slowly as it was raised, each of the movements described above being performed in reverse order, the whole by the sheer force of muscular energy, in opposition to the gravity of the dumb-bell. Special care should be taken as before, not to find support for the free arm on the knee, thigh, hip, or flank of its side of the body. When the dumb–bell again rests on the floor, a similar series of movements is practiced by the other hand and arm, the body bending as before, forward and downward, to reach the dumb-bell, upward to the erect position as the weight is carried to the side, forward and downward again as the weight is again brought to the ground. In this way all the muscles of the body are gradually and systematically brought into play, in a gentle exercise which does not share the violence required by the more sudden demands made, for example, in leaping, lifting, or the exercises of the trapeze. By raising in this way six thousand pounds to a height of nearly six feet in about one hour, a man of average height, weight, and strength, can, in a temperature of 60° F., induce an agreeable perspiration, and a thrill of activity in every part of his frame, which will, if the exercise be repeated regularly day after day, add very greatly to the weight and vigor of every part of his body. It is of course understood that this is merely suggested as a substitute for the invigorating exercises of out-door life, both in the form of labor and amusement, from which so many residents of towns and cities are perforce restricted.

No less systematic should be the practice of massage of the abdomen, should such be required. For this purpose the body should rest in a reclining position, in order to relax the abdominal muscles. The operation should not be performed fitfully or hastily. A given hour each day, preferably in the evening before seeking rest, should be sacredly devoted to the purpose. Its limits should on each occasion be fixed by a time-piece. The entire abdominal surface should then be regularly kneaded ; not rubbed, but impressed with a series of alternate strokes of the hands at first upward in the direction of the colon, then from right to left, and afterward downward, the whole to be concluded with a series of compressive movements in which the abdomen is grasped by the two hands and gently swayed in a twisting or rocking motion from side to side. If one can here have the aid of an accomplished masseur, or masseuse, the end is, without doubt, more perfectly attained ; but in their absence, they who really need such service should be urged to practice the operation regularly and systematically upon themselves. In thousands of cases have patients, under the direction of a competent physician, received marvelous benefits by the aid of their own hands in rubbing, kneading, and othewise manipulating various parts of their own bodies.

With respect to the selection of foods which are most suitable and least objectionable in each case, it should be remembered that errors are committed in widely divergent directions. The so-called "health-foods," such as oat-meal, cracked wheat, etc., are, without question, useful in certain cases, but when the victim of dyspepsia or habitual constipation has once determined to support existence chiefly upon this dismal sort of diet, he will surely be worse before he is better. These articles are often positively irritating to the mucous lining of the digestive canal, which, by reason of a condition of torpidity, of anæmia, or of muscular weakness, is often totally unequal to the task of converting, for example, a bowlful of "wheaten grits" into protoplasm. It should never be forgotten that man requires a mixed diet; that to digest his food well it must be made attractive to his palate, and that it is always better assimilated when it is set before him in a state which is attractive to the eye. No hard and fast lines can here be drawn. Too many persons, living in the shadow of fear less they commit dietary mistakes, or in consequence of a languid disinclination for food resulting from their sedentary habits, eat an insufficient amount of food. This is particularly true of women who lead indolent lives. They rise in the morning—for breakfast, swallow a cup of coffee and a delicate slice of buttered toast; they have no appetite for more than this. At luncheon, or at supper, they take a cup of tea, with toast or bread, and a thin slice of cold fowl or warmed beef, to which possibly a fragment of cake or a small quantity of "sweets" is added. At dinner, they partake very sparingly of meat with potato, or some other vegetable, and some trifle for dessert.

Now, this is a diet-list almost of the character which might be recommended to a patient affected with looseness of the bowels; the tea and toast or bread, which constitute so large a part of the dietary of too many women, being admirably adapted to beget and sustain a condition of habitual constipation. Persons accustomed to this wretchedly meager supply of food, need, first, an amount of exercise sufficient to cause a demand for more aliment; and, second, a larger quantity of the latter, in proportions adapted to the manifold characters of the appetite and the increasing digestive capacity. In proportion as the bowel is distended with a digestible mass of chyme, elaborated from a judiciously commingled animal and vegetable food, will it be able to recognize its natural and healthy stimulus to contraction, and to actively obey it.

The laxative effect of fruit is pretty generally recognized by the laity, but when asked whether they are using fruit for the purpose of securing relief from habitual constipation, they will often respond that this is the case, when the actual consumption of the same is quite insufficient for the purpose in view. A small quantity of fruit, consumed after a dinner of meats, is not enough to secure a laxative effect in the really constipated. The improved railway communication of this country has brought the fruit markets of the tropic and temperate zones into such proximity, that now there is scarcely a day in the year when the resident of the larger towns can not at very moderate rates supply himself with excellent fruit of wholesome quality. This should be regularly purchased and systematically eaten by the persons who are in greatest need of it, and at the hours when the stomach is empty. Thus, the fig, the orange, the apple,may be employed on rising in the morning, almost exclusively for the luncheon at noon, and often, by those who like them, even at night before going to rest, popular prejudice to the contrary notwithstanding. The chief point to be noted in this connection is, not that fruit has this effect, which many very ignorant people understand quite well, but that persons requiring a diet of fruit sufficient to move the bowels need to be encouraged by their physicians to systematically frequent the fruit markets,to purchase for themselves the best and freshest fruits to be had, not for the table exclusively, but for their individual purposes, in the bed room or the dressing-room, and that such purchases should be intelligently and systematically devoted to securing the best results.

In this connection it is scarcely necessary to add, that a glass or more of pure water, swallowed on an empty stomach before breakfast, or at night before seeking the bed, or on both occasions, is often in itself sufficient to secure a satisfactory motion. It is a common prejudice that water drinking, even among those who pride themselves upon temperance in alcoholic stimulants, is an evil practice. It is difficult to understand how this strange infatuation originated. The instinct for pure water is acknowledged by all the vertebrates; and the largest consumers of it are often the longest lived and best nourished. The Arab of the desert points with pride and admiration to his fleet Arabian courser dipping his muzzle into the stream to the level of his eyes as he drinks, and claims that in this way his horse proves the excellence of his breeding. Certainly many persons of no pretensions to scientific acquirements have again and again remarked that those who consume a large quantity of water with their meals are usually better nourished and more free from digestive troubles than are others. Perhaps it is the loading of the stomach with hurtful and even dangerous draughts of iced or unduly cooled water that has given rise to the prejudice. Whatever may have been its origin, it should be relegated to the old women and the occupants of the nursery, as unworthy of other company. Those suffering from indigestion and constipation are always better for drinking freely of pure water at a temperature sufficiently cool to be merely acceptable to the palate. It is of course presumed that this fluid is potable. That which is suspected should be always filtered; and the better filters are cheaper in the end, than the purchase of the various spring waters, which, however excellent in their purity, have to be brought from a distance in casks lined with paraffine, and to be thus sold at rates prohibiting their largest employment.

Among the residents of regions in the West where the "hard" and lime waters are chiefly used for purposes of the table and the kitchen, there is a source of relief for the constipated which is quite too often overlooked. I refer to the use of rain water for drinking and cooking purposes. The erection of carefully constructed cisterns is the chief need of many of the smaller towns in the West; for the use of rain water, when it is cleansed of impurities by filtering, will often solve the entire question, the persons using it having no further need of cathartics of any description.

The consideration of this important subject would be quite imperfect, if no reference were had to the need of regularity in the time of evacuating the bowels. Upon this point, as upon others to which there has been occasion to refer, women are sinners above others. With men, the pressure of business demands and the limitation of time have in many cases begotten and enforced attention to this great requisite. The hour after breakfast, or some other meal, is set aside for the daily evacuation of the bowels. There is with many men a sense of discomfort when the needed relief is not thus had, and often a corresponding precaution as to the morrow.

Unfortunately, to many women the associations of the temple of Cloacina and the oblations required by that goddess are, foolishly enough, extremely repugnant. They defer to an emergency, or to an imperative call, the duty of regularly evacuating the rectum, many of them hastily visiting the closet, merely to leave there the fæces immediately impinging upon the inner sphincter of the bowel. It is, of course, needless to say here that a reform in such matters is absolutely indispensable to a correction of the constipated condition.

As an illustration of the advantage which plain speaking and common sense possess over all sorts of false modesty and pseudo-"refinement," I am tempted to refer to a well known incident in the life of one of the most scholarly and accomplished physicians of this country, who not long since passed to his long rest with the respect and esteem of all his peers. He was about to accompany a large party of gentlemen and ladies on an extended tour through Europe, and before going on board of the steamer he collected the latter in one of the reception rooms of a hotel in New York, and thus addressed them: "We are about," he said, "to make a long journey together, as you all well know, and it becomes me, not as the physician of the party, for we need none such, but as a physician, to give you a word of advice and warning as to my course. I wish to see you all return to this country in just as good health as you are at this moment, even better; and in the meantime to be able to enjoy foreign travel without the vexatious and disappointing delays incidental to temporary attacks of illness on the part of any one, which might thus interfere with the plans and the pleasure of the entire party. With this end in view, I desire to declare in the most positive manner, that I shall refuse to start with the party on a day's journey until every member of it has had a satisfactory movement of the bowels. As your sex is most negligent of this important duty, I shall enforce the rule particularly with you." The interesting point of the anecdote is that all the travelers returned to the city of New York in sound health, no one of them during a year's absence having been ill a single day.

The need of regularly attending to the movement of the bowels is misinterpreted by some. They deem this need to rest upon the danger of merely forgetting the hour set apart for this necessary duty. But this is the most imperfect view of the case. The need is really based upon, first, the possibility of attaining high results by training the body; second, the law that regularity of departure, with uninterrupted transmission, involves regularity of arrival.

Few realize the degree to which human bodies are in process of daily training; and fewer yet the excellence to which such training can be brought. As regards the voluntary muscles, a good violin or piano-player furnishes an excellent illustration of their perfect training. As to the involuntary muscles, or even those which, being voluntary, are involuntarily employed, it is scarcely necessary to remind medical men that individuals have been found who could shift the position of the heart, dilate the pupil, regurgitate food from the stomach, and even empty the uterus at will. Exceptional as these facts must necessarily be, they are yet in a high degree instructive. I was recently shown, in a tack factory, a workman apparently asleep, seated before a machine which was engaged in chewing a slip of metal into myriads of tacks. It was his duty to present first one side, then the other, of the metallic strip, to the insatiable jaws of the iron monster in his presence, the motion being alternated, I should say, about once every two seconds. Long practice had so accustomed him to this work that, even in sleep, his voluntary muscles performed this delicate task when he was quite unconscious of their movement. Even a puppy but a few weeks old, then little more than a mass of animated protoplasm, has been taught to perform tricks.

Now, a man's or a woman's bowel can be taught better tricks than these by any one who determines upon it. It is a gross mistake to look upon this sentient and complex organ as though it were nothing more than a metallic waste pipe. He who accustoms his bowel to moving after the morning meal, or at another selected hour, trains every muscle connected with the expulsive act to perform its part. The bowel can be trained as well as the finger or the hand. I have known a physician who regularly had a stool soon after he was summoned at night to an obstetric call, a practice which he adopted early in his professional career, in order to avoid trouble which once befell him when in attendance upon a tedious case. At that time he could not escape from the bedside of a woman in labor for hours after he had been suffering distress,and it was this experience which had induced him to acquire the habit. One of my own patients, having similarly suffered at a social gathering, established a habit which required him to go to stool as soon as he had put on his evening dress coat —an uncomfortable trick, from which, as he grew older, he could with difficulty escape. Any one who has had occasion to watch, with careful attention, the habits of the domesticated lower animals, has noticed how regularly they attend to the calls of nature, and at periods, too, when most conducive to their comfort. Thus a horse will often stale regularly before his bed is made for the night, lest otherwise it be made uncomfortable for him ; and the first out-going in the morning, from the air of the stable to that of the outer world, will time his morning movement. It is needless to say to one who has given any attention to the subject that patience, aided by a little wisdom, can train the most rebellious bowel to the needs of the constipated.

And the second point, to which I have referred, should not be overlooked. Every train-dispatcher of a railway organization understands that the regularity with which his engines and cars reach their destination is proportioned to the regularity with which they are dispatched over the road. And so, when the food is regularly, (usually three times in the day,) made to enter the upper part of the alimentary canal, if the digestive activity be not interrupted, there will regularly arrive at the lower part the débris left by the complicated process to which it has been subjected. If then this lower part be regularly emptied, it follows that the entire transit from end to end will be liable to fewer interruptions, and be thus in other ways more perfectly accomplished. On the contrary, if the food regularly enters the upper part, and the debris be only irregularly removed from its lower part, it follows that the transit from end to end will be correspondingly less regular, and disturbances of all kinds be, as a consequence, much more readily induced. It cannot, in brief, be denied that mere feculent accumulation in the rectum exercises a potent influence high up in the alimentary canal. He who argues otherwise has the most imperfect knowledge of the human economy.

Thackeray, in some clever verses which describe a little page, sighing and singing beneath the window of his sweetheart, Bonnie Bell, cries:—

"Wait till you come to forty year!"

It is rarely before the fortieth year of life that the need, the importance, and the value of a satisfactory movement of the bowels each day, is fully appreciated. Happy the young man or the young woman who, as to this matter, receiving well-timed counsel from the lips of a wise physician, is careful to observe the same through all the years of advancing and maturing age. Happy also the physician who, with a brain enriched with the treasures of pathology, the wealth gathered by the aid of the microscope, and the ore mined from a crude materia medica, has the prudence and judgment to know in what cases their precepts must be interpreted in the light of a profound study of the social habits of the people among whom he moves; a physician so high-hearted that the loss of the few shillings he might gain, by ordering drugs in a prescription, is more than weighted by a conscientious desire to meet the real physical needs of those who come to him for help. Happy, too, that commonwealth whose physicians are esteemed, yea, and well rewarded also, for a wisdom that is somewhat larger than a dispensatory, and stronger than to ask the aid of an apothecary in achieving its highest ends.

---

The Chicago Throat and Chest Hospital received a set of laryngeal instruments from Messrs. Sharp and Smith as a Christmas gift.

